# Comparing affective bias modification by first- and second-generation antidepressants in male rats using a translational behavioural task

**DOI:** 10.1038/s41386-026-02376-4

**Published:** 2026-02-26

**Authors:** Katie A. Kamenish, Emma N. Cahill, Emma S. J. Robinson

**Affiliations:** University of Bristol, School of Physiology, Pharmacology and Neuroscience, Bristol, UK

**Keywords:** Depression, Cognitive neuroscience

## Abstract

Negative affective biases influence cognitive and emotional behaviours and have been observed in patients with major depressive disorder. The neuropsychological hypothesis of antidepressant efficacy suggests direct modulation of affective biases may contribute to efficacy. Studies have shown conventional antidepressants can positively bias emotional processing following acute administration in humans. This study employs a rat model developed to study affective biases based on associative learning and memory, and used this assay to compare selected first versus second generation antidepressants. Dose-response experiments using the tricyclic antidepressant, amitriptyline (0.3–3.0 mg/kg), monoamine oxidase inhibitor, moclobemide (3.0–10.0 mg/kg) and serotonin specific re-uptake inhibitor, sertraline (1.0–10.0 mg/kg) were performed. Specific protocols permitted quantification of affective biases associated with new learning or the acute and sustained modulation of past experiences. All treatments positively biased new learning but exhibited differences in terms of their ability to modulate negatively biased memories. Amitriptyline and sertraline attenuated negatively biases memories when administered 1 h or 24 h before testing. Moclobemide had no effects on past experiences. No treatments had effects in the control reward learning assay. Although generally considered to have similar efficacy and time course of effects, the pharmacological profiles of these antidepressants differ. Previous work has shown that variations in affective bias modification are linked to both the time course of clinical effects and their interaction with experience-dependent plasticity. Integrating understanding of these neuropsychological differences within clinical practice has the potential to improve clinical outcomes for patients.

## Introduction

First- and second-generation conventional antidepressants (CADs) enhance monoaminergic neurotransmission [1] and include the tricyclic antidepressants (TCAs) [2], monoamine oxidase inhibitors (MAOIs) [3], and selective serotonin and/or noradrenaline reuptake inhibitors (SSRIs) [4]. Conventional antidepressants are generally thought to mediate their antidepressant effects via similar downstream mechanisms, but all have distinct pharmacological profiles that may carry clinical relevance [5–7]. The discovery of first-generation antidepressants was largely serendipitous [8], but subsequent characterisation of their pharmacology, together with evidence implicating serotonin and noradrenaline in major depressive disorder (MDD) [1, 9], informed the development of second-generation antidepressants [10]. These drugs act more specifically on these monoaminergic targets, thereby improving safety and reducing side-effect burden e.g. SSRIs lack the anticholinergic side effects of TCAs [4, 6]. However, recent studies suggest that muscarinic receptor antagonism, such as that produced by scopolamine, elicits rapid-acting antidepressant effects (RAAD) [11, 12]. It is widely accepted that all CADs have a delayed onset of mood improvement, typically between four and six weeks [13, 14]. However, early clinical observations of the TCA imipramine suggested the emergence of beneficial effects within days [8]. These findings raise the possibility that muscarinic antagonism associated with TCAs may contribute to their therapeutic effects [6], and that pharmacological differences among CADs could influence their neuropsychological effects and temporal dynamics for clinical outcomes [15].

A key challenge in studying antidepressant mechanisms is the widespread reliance on self-reported symptoms [1], which lack specificity and sensitivity compared to traditional biomarkers such as blood assays or neuroimaging [5]. Rather than focusing on DSM-based diagnostic criteria, the Research Domain Criteria (RDoC) framework emphasizes symptom domains and the relationship between neurobiology and behaviour through objective measures [16]. Considering MDD within this framework, negative affective biases represent a potential trans-diagnostic ‘behavioural biomarker’ for quantifying emotional symptoms in psychiatry. Affective biases are an important neuropsychological mechanism that modulate cognition and emotional processing. Negative biases are defined as the preferential tendency to attend to, interpret, or recall negative over positive information [17–19], and have been linked to the reinforcing maladaptive cognitive patterns seen in MDD [9]. According to this neuropsychological model, it is hypothesised that CADs exert their therapeutic effects by shifting cognitive-affective processing toward a more positive valence, which subsequently influences experience-dependent plasticity and mood [5, 20–22]. The delayed onset of action is attributed to the time required for new, positively biased experiences to accumulate sufficiently to produce a subjective change in mood [23–25]. By contrast, the RAAD ketamine improves mood within 24 h of a single acute dose, with effects sustained for up to 14 days [26, 27], suggesting the mechanisms underlying its antidepressant effects differ from CADs [28]. Although not yet empirically tested in humans, one emerging hypothesis is that RAADs modulate affective biases associated with past experiences, and neuropsychological differences may underlie the distinct time course of their clinical effects [24, 29].

The Affective Bias Test (ABT) and Reward Learning Assay (RLA) are associative learning tasks adapted from neuropsychological tasks used in humans to quantify affective and reward-induced biases, respectively [30, 31]. In the ABT protocol, two independent cue-reward memories of equal value are generated under either control or treatment conditions. A subsequent choice test is then used to quantify any arising affective state-induced biases. This protocol can be adapted to distinguish whether treatments modify affective biases associated with new learning (via pre-encoding administration) or modulate the retrieval of a negatively biased memory (via pre-choice test administration) with either an acute (<1 h pre-treatment) or a sustained effect (>24 h pre-treatment) [29, 31]. The ABT can therefore be used to investigate different types of affective bias modification and compare the effects with clinical findings in terms of efficacy, rate of onset and whether the effects endure beyond acute drug exposure. The RLA is used as a control memory retrieval task to determine whether effects observed in the ABT reflect specific modulation of affective bias rather than generalised learning impairments. Previous studies have found distinct neuropsychological effects associated with CADs and RAADs. CADs positively bias new experiences while RAADs modulate negatively biased memories when administered either 1 h or 24 h before the choice test [24, 29]. Furthermore, both ketamine and the psychedelic psilocybin facilitate re-learning of the negatively biased memory with a relatively more positive affective valence 24 h post-treatment [29]. These effects on past experiences are not seen with the mixed re-uptake inhibitor venlafaxine, suggesting CADs only bias new experiences, an effect which may explain their delayed onset of action [24, 29, 30].

The present study investigated the effects of different classes of CADs in male rats using the three different ABT protocols to compare their effects on new learning and acute and sustained modulation of negatively biased memories. To control for any general impairments in learning or retrieval, we included the RLA. Previous studies suggest antidepressant effects are specific to modulation of affective-state induced biases and have no effect on reward-induced biases. However, treatments which disrupt learning and memory impair performance in both the ABT and RLA. Specifically, the tricyclic antidepressant amitriptyline, the monoamine oxidase inhibitor moclobemide, and the selective serotonin reuptake inhibitor sertraline were compared using a new learning protocol and post-learning modulation of a negative affective bias, administering treatment either <2h or 24 h prior to the choice test. All drugs were tested across a dose range predicted to encompass clinically relevant levels of receptor occupancy.

## Materials and methods

### Animals & housing

Three separate cohorts of male Lister Hooded rats (Charles River, UK; Envigo, UK) were used in these experiments (*n* = 11–16 per group; Supplementary Table S4). Since previous work reported no notable sex differences regarding drug effects in the ABT, this study only used male rats [32, 33]. Animals were pair-housed in standard enriched laboratory cages (shelter, cardboard tube, cotton rope and chew block) under a 12:12 h reverse light-dark cycle (lights off: 08:00 h, lights on: 20:00 h) and temperature-controlled conditions (21 ± 1 °C). Rats were food restricted to ~90% of their free-feeding weights, aligned with the normal growth curve for male Lister Hooded rats maintained at a normal-to-overweight body condition score. Water was provided ad libitum. All experimental procedures occurred during the animals’ active phase (09:00 h and 16:00 h). Animals were tested in either the ABT or RLA assay following systemic administration of one of three CADs: amitriptyline, moclobemide, or sertraline. Each drug was tested in a separate experiment, and all experiments employed a fully randomised, within-subject design where animals received all treatments. Experiments randomised potential confounds including substrate, pairing session and treatment. Further details of the ABT/RLA protocols and a list of substrates can be found in the supplementary materials (Supplementary Fig. S1 (created in Biorender), Supplementary Tables S1–3). Researchers were blinded to treatment for each experiment. No subjects were excluded from data analysis. All procedures adhered to the UK Animals (Scientific Procedures) Act 1986 and under a project license from the UK Home Office. Ethical approval was granted by the University of Bristol Animal Welfare and Ethical Review Body.

### General protocol

#### Training

The experiments discussed here followed the same training protocol outlined in Hinchcliffe et al. [27] using the same apparatus (Supplementary Fig. S1). Rats began training by learning to dig in two identical ceramic bowls containing sawdust with the level of difficulty increasing incrementally over five days. On Day 5 of training, animals completed a discrimination session using two novel substrates to confirm they had sufficiently learned the key rule of the task – that digging in the correct substrate resulted in a food reward. ‘Correct’ trials were those in which the animal chose the bowl containing the reward-paired substrate, and ‘incorrect’ trials when they chose the unrewarded substrate. An ‘omission’ was recorded if an animal failed to approach and/or explore the bowls within 30 s. Training was complete once the animal achieved six consecutive ‘correct’ trials in the discrimination session.

### Affective bias test

The ABT experiments followed a standard protocol each week [30] composed of four pairing sessions (one session per day) during which animals generate independent cue-specific memories for two reward-paired substrates (Supplementary Fig. S1). In the first pairing session, the rat was presented with one bowl containing a food reward-paired digging substrate (substrate ‘A’) and another bowl containing a distinct, unrewarded ‘blank’ substrate. The pairing session concluded when the rat had completed six consecutive ‘correct’ trials. The second pairing session followed the same protocol but with a different reward-paired substrate (substrate ‘B’). Both pairing sessions were repeated so that animals underwent four pairing sessions each week over consecutive days. Each animal learnt one substrate-reward association under drug treatment and the other under vehicle conditions. On day 5 (or day 6 for sustained effects experiments), retrieval of the cue-specific memories was tested either with (Supplementary Fig. S1a–d) or without drug pre-treatment (Supplementary Fig. S1a). To quantify the arising affective or reward-induced bias, animals underwent a 30-trial choice test where both previously rewarded substrates (‘A’ and ‘B’) are presented, with each substrate having a 1-in-3 reward probability under a random reinforcement schedule. The number of choices per substrate, response latency per trial, and omitted trials were recorded.

### Reward learning assay

The RLA follows a similar protocol to the ABT with four pairing sessions followed by a choice test, except the animals’ affective state remains the same throughout the five-day protocol [30]. Instead, animals learn to associate one substrate with a high-value reward (two reward pellets) and the other with a low-value reward (one pellet) (Supplementary Fig. S1c). Drug treatments were administered before the choice test. Only treatments which induced an acute attenuation of negative affective bias (Supplementary Fig. S1b) were tested using the RLA.

### Drugs

The tricyclic antidepressant, amitriptyline, reversible monoamine oxidase A inhibitor, moclobemide and serotonin re-uptake inhibitor, sertraline were tested in the three different ABT protocols. The mixed serotonin and noradrenaline re-uptake inhibitor, venlafaxine, was included as a positive control. FG7142 was used to induce a negative affective bias for studies testing treatment effects on biased memories. Details of the drug doses, pre-treatment times, routes of administration and source are detailed below (Table 1).

**Table 1:** Summary of drug treatment conditions used in affective bias and retrieval experiments.

	Route	Dose (mg/kg)	Pretreatment	Experimental use
Amitriptyline (tricyclic antidepressant; Sigma-Aldrich, UK)	p.o.	0.3, 1, 3	*t* = −2 h	New learning: all doses tested. Retrieval: doses producing a positive affective bias (0.3, 1, 3 mg/kg) administered 2 h and/or 24 h prior to the choice test following FG7142-induced negative bias. RLA: 0.3 and 1 mg/kg tested for specificity.
Moclobemide (reversible MAO-A inhibitor; Tocris Bioscience, UK)	s.c.	3, 10	*t* = −30 min	New learning: both doses tested. Retrieval: doses producing a positive affective bias (3, 10 mg/kg) administered 30 min and/or 24 h prior to the choice test following FG7142-induced negative bias.
Sertraline (SSRI; Sigma-Aldrich, UK)	p.o.	1, 3, 10	*t* = −2 h	New learning: all doses tested. Retrieval: doses producing a positive affective bias (3, 10 mg/kg) administered 1 h and/or 24 h prior to the choice test following FG7142-induced negative bias. RLA: 3 mg/kg tested for specificity.
Venlafaxine (SNRI positive control; LKT Laboratories, UK)	p.o.	3	*t* = −2 h	New learning only.
FG7142 (benzodiazepine inverse agonist; Sigma-Aldrich, UK)	s.c.	3	*t* = −30 min	Retrieval context: administered prior to pairing sessions to induce a negative affective bias.

*ABT* affective bias test, *RLA* reward learning assay, *p.o.* per os (oral), *s.c.* subcutaneous, *t* time prior to behavioural testing.

For voluntary oral dosing, animals were trained to drink from a 1 ml syringe prior to start of experiments [34]. Both amitriptyline and venlafaxine were prepared in a solution of 10% condensed milk and 0.9% saline (1 ml of condensed milk per 10 ml solution). Sertraline was prepared in a solution of 5% DMSO and 95% peanut butter/sunflower oil mixture and administered via voluntary ingestion. Moclobemide was dissolved in 0.9% sterile saline and injected subcutaneously due to issues maintaining compliance to oral dosing. Injections were performed using techniques requiring minimal restraint of the animal and involving injection into the left or right flank (alternated daily). Drug solutions for all experiments were freshly prepared on each day of treatment and administered in a 1 ml/kg dose volume.

### Statistical analysis

Data were analysed using SPSS Statistics 29 and figures were created using GraphPad Prism 10.2.2 (GraphPad Software, USA). Choice bias was calculated as the number of choices for the drug-paired substrate (ABT) or two pellets-paired substrate (RLA) divided by the total number of trials (30). The result was multiplied by 100 to generate a % choice, with 50 subtracted to yield a final score % bias score. The magnitude of bias generated in the ABT and RLA are relatively small as absolute values, however, the effect size is large due to low between and within-subject variability (effect size 1.0–1.2, [33]). The positive and negative affective biases generated in the ABT are also similar in magnitude to those seen in human subjects performing objective behavioural tasks designed to quantify biases in emotional processing [23]. Values exceeding two standard deviations from the cohort mean were excluded. Data from animals completing fewer than 15 choice test trials were also removed. Choice bias and response latency scores during the choice test were analysed using repeated measures ANOVA (RM-ANOVA) with ‘TREATMENT’ as the within-subject factor. Pairwise comparisons were conducted via two-tailed paired t-tests or Dunnett’s test when post-hoc analysis was required. Affective biases within treatment groups were tested using a one-sample t-test against a null hypothesis of 0% choice bias. Comparisons to vehicle were used to assess drug effects relative to control conditions, whereas one-sample tests for each treatment alone was compared against 0% bias to determine if there was a significant positive or negative affective bias. Each animal’s mean trials to criterion, latency to dig, and omitted trials during pairing sessions were analysed using RM-ANOVA or a two-tailed paired *t*-test. Choice test data were further analysed to assess non-specific effects (e.g., sedation, increased urination/defecation). Additional *post-hoc* comparisons were performed using two-tailed paired *t*-tests between control (vehicle/low-value reward) and treatment/manipulation (drug treatment/high-value reward) for each week. Shapiro–Wilk tests assessed normality for % choice bias, trials to criterion, and mean latency to dig. Mauchly’s sphericity test was used to validate a RM-ANOVA. Effect sizes are presented as Cohen’s *d* for t-tests and post-hoc tests, or as η2 for RM-ANOVA.

## Results

### Experiment 1: Conventional antidepressants positively bias new reward-associated memories

**Fig. 1:**
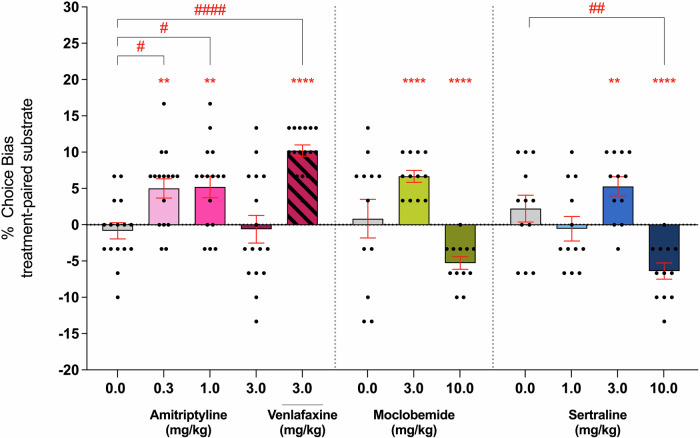
CAD treatment produced a dose-dependent affective bias, with low doses inducing a positive bias and high doses either having no effect or inducing a negative bias. Rats were administered amitriptyline (0.3, 1, and 3 mg/kg; *n* = 16) or venlafaxine (3 mg/kg; *n* = 16), moclobemide (3 and 10 mg/kg; *n* = 12), or sertraline (1, 3, and 10 mg/kg; *n* = 12) before substrate-reward pairing sessions. Choice bias was assessed 24 h after the final pairing session. Amitriptyline (0.3 and 1 mg/kg) induced a significant positive bias, comparable to venlafaxine (3 mg/kg), *F*₄,₆₀ = 10.82, *p* < 0.0001). Moclobemide (*F*₂,₂₂ = 11.84, *p* = 0.0029) and sertraline (*F*₃,₃₃ = 9.259, *p* = 0.0016) both showed significant effects, with 3 mg/kg inducing a positive bias and 10 mg/kg inducing a negative bias, though only the highest dose of sertraline significantly differed from vehicle (*p* = 0.0069). Data are shown as mean % choice bias ± SEM and were analysed using a one-sample *t*-test (^*^p < 0.05, ^**^p < 0.01, ^***^p < 0.001) and Dunnett’s test following main effect with RM-ANOVA (^#^*p* < 0.05, ^##^*p* < 0.01, ^####^*p* < 0.0001).

All three conventional antidepressants positively biased new learning at specific doses, though with dose-dependent variability (Fig. 1). Low-dose amitriptyline (0.3 & 1 mg/kg) induced a positive bias toward the treatment-paired substrate similar to the bias produced by the positive control, 3 mg/kg venlafaxine treatment (*F*_(4,60)_ = 10.82, *p* < 0.0001, *η*^2^ = 0.419, *n* = 16). The highest dose of amitriptyline (3 mg/kg) had no effect versus the vehicle treated group. There was a main effect of treatment with moclobemide (*F*_(2,22)_ = 11.84, *p* = 0.0029, *η*^2^ = 0.518, *n* = 12), and a positive affective bias was observed at the 3 mg/kg dose (one-sample *t*-test, *t*_(11)_ = 8.124, *p* < 0.0001) but this was not different from control in the pairwise comparison (Dunnett’s test, *p* = 0.1176). The 10 mg/kg dose induced a negative affective bias (one-sample *t*-test, *t*_(11)_ = 6.902, *p* < 0.0001) but was also not different from control in the pairwise comparison (Dunnett’s test, *p* = 0.1064). A similar effect was seen with sertraline with a main effect of treatment (*F*_(3,33)_ = 9.259, *p* = 0.0016, *η*^2^ = 0.457, *n* = 12). A positive bias at 3 mg/kg (one-sample *t*-test, *t*_(11)_ = 3.978, *p* = 0.0022) and negative bias at 10 mg/kg (one-sample *t*-test, *t*_(11)_ = 5.702, *p* < 0.0001) were observed but only 10 mg/kg produced a bias significantly different from vehicle in the *post-hoc* analysis (Dunnett’s test, *p* = 0.0069). Neither amitriptyline nor sertraline produced any non-specific effects during pairing sessions, but rats administered 10 mg/kg moclobemide exhibited slower latency to respond (*F*_(2,22)_ = 23.29, *p* < 0.0001) and more trial omissions (*F*_(2,22)_ = 6.280, *p* = 0.0070) compared to the vehicle group (Supplementary Fig. S2, Supplementary Table S5).

### Experiment 2: Conventional antidepressants differentially modulate memory-specific negative affective biases

**Fig. 2:**
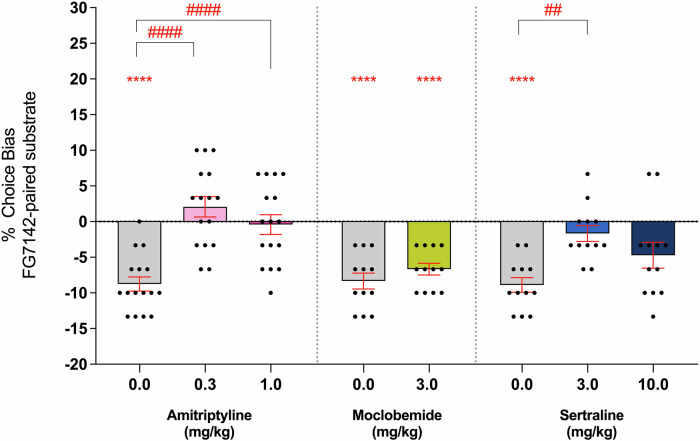
Memory-specific negative affective biases are attenuated following treatment with amitriptyline and sertraline but not moclobemide. FG7142 (3 mg/kg) was administered before substrate-reward pairing sessions to induce a negative affective bias, and rats were then treated with amitriptyline (0.3 and 1 mg/kg, *n* = 16), moclobemide (3 mg/kg, *n* = 12) or sertraline (3 and 10 mg/kg, *n* = 12) prior to choice testing. Amitriptyline attenuated the FG7142-induced negative affective bias (*F*_(2,30)_ = 23.43, *p* < 0.0001), with no bias observed at both 0.3 mg/kg (*t*_(15)_ = 1.464, *p* = 0.1639) and 1 mg/kg (*t*_(15)_ = 0.2997, *p* = 0.7685). Sertraline showed a similar attenuation during choice testing (*F*_(2,22)_ = 6.652, *p* = 0.0055) but only at the lowest dose (*p* = 0.0028), and animals given 10 mg/kg continued show a negative affective bias (*t*_(11)_ = 2.609, *p* = 0.0243). Moclobemide had no attenuating effect (*t*_(11)_ = 1.000, *p* = 0.3388), with negative bias persisting at 3 mg/kg, *t*_(11)_ = 7.416, *p* < 0.0001). Data are shown as mean % choice bias ± SEM and were analysed using a one-sample *t*-test (^*^p < 0.05, ^****^p < 0.0001) and Dunnett’s test following main effect with RM-ANOVA or two-tailed paired *t*-test (^##^*p* < 0.01, ^####^*p* < 0.0001).

Amitriptyline and sertraline, but not moclobemide, attenuated the FG7142-induced negative bias when administered <2 h before testing (Fig. 2). The FG7142-induced negative bias was attenuated in rats pre-treated with either 0.3 or 1 mg/kg amitriptyline 2 h before the choice test (*F*_(2,30)_ = 23.43, *p* < 0.0001, *η*^2^ = 0.591, *n* = 16). There was no bias observed for both 0.3 mg/kg (one-sample *t*-test, *t*_(15)_ = 1.464, *p* = 0.1639) and 1 mg/kg amitriptyline (one-sample *t*-test, *t*_(15)_ = 0.2997, *p* = 0.7685) when compared against a neutral 0% choice bias. Sertraline pre-treatment also attenuated the FG7142-induced negative bias with a main effect of treatment (*F*_(2,22)_ = 6.652, *p* = 0.0055, *η*^2^ = 0.591, *n* = 12). No bias observed at 3 mg/kg (Dunnett’s test, *p* = 0.0028, one-sample *t*-test, *t*_(11)_ = 1.483, *p* = 0.1661), but a negative bias was still seen following 10 mg/kg (one-sample *t*-test, *t*_(11)_ = 2.609, *p* = 0.0243). Moclobemide treatment had no acute effects on retrieval (two-tailed paired *t*-test, *t*_(11)_ = 1.000, *p* = 0.3388, *d* = 3.41, *n* = 12). None of the treatments or doses tested showed any non-specific effects during the choice test, with similar latencies and numbers of omissions observed in all groups (*F* < 2.89, *p* > 0.05) (Supplementary Table S6a). Treatment with FG7142 also did not produce any such effects on performance during pairing sessions (Supplementary Table S6b).

### Experiment 3: Acute effects of amitriptyline and sertraline on negative affective biases are not result of non-specific impairments in memory retrieval

**Fig. 3:**
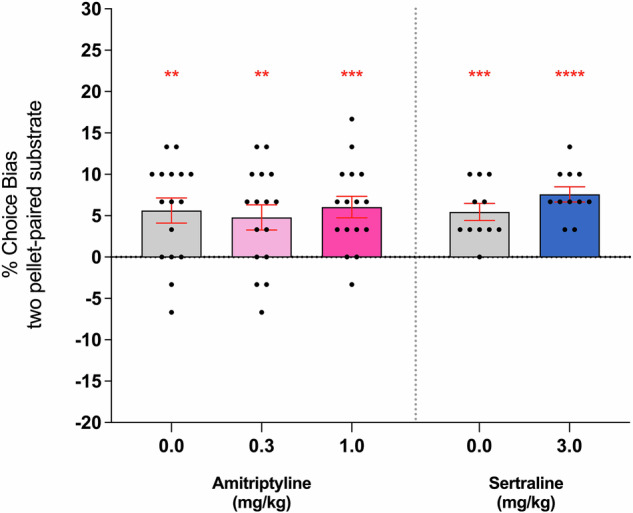
Amitriptyline and sertraline did not impair reward learning or retrieval of reward-associated memories in the RLA, confirming that the effects on affective bias observed in Experiments 1 and 2 are not due to non-specific impairments of learning and/or memory. A reward-induced positive bias was induced using high-value (two pellets) versus low-value (one pellet) reward pairing sessions, and rats were administered amitriptyline (0.3 and 1 mg/kg, *n* = 16) or sertraline (3 mg/kg, *n* = 11) prior to choice testing. Both amitriptyline (*F*_(2,30)_ = 0.2381, *p* = 0.7896) and sertraline (*t*_(10)_ = 1.295, *p* = 0.2243) failed to disrupt the positive bias toward the high-value reward substrate, with animals demonstrating a reward-induced bias similar to that of the vehicle-treated controls. Data are shown as mean % choice bias ± SEM and were analysed using a one-sample *t*-test (^**^p < 0.01, ^***^p < 0.001, ^****^p < 0.0001) and Dunnett’s test following main effect with RM-ANOVA or two-tailed paired *t*-test.

Neither amitriptyline nor sertraline affected reward-induced bias in the RLA, confirming specificity to affective processing (Fig. 3). Animals treated with vehicle prior to the choice test exhibited the reward-induced positive bias consistent with previous studies using the RLA (*F* < 3.32, *p* > 0.05). A similar reward-induced bias was observed in animals treated with both amitriptyline (*F*_(2,30)_ = 0.2381, *p* = 0.7896, *n* = 16) and sertraline (two-tailed paired *t*-test, *t*_(10)_ = 1.295, *p* = 0.2243, *n* = 11), indicating that the attenuating effects of both drugs reported in Experiment 2 are specific to affective bias modulation. None of the treatments or doses tested in the RLA produced any non-specific effects during the choice test (Supplementary Table S7*)*.

### Experiment 4: Amitriptyline and sertraline show sustained modulation of negative affective biases 24 h following treatment

**Fig. 4:**
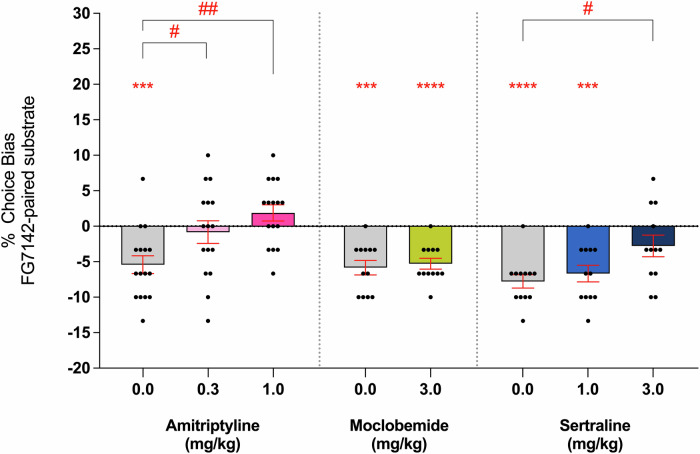
CAD-induced modulation of negative affective bias is sustained for 24 h post-treatment. FG7142 (3 mg/kg) was administered before substrate-reward pairing sessions to induce a negative affective bias, and rats were then treated with amitriptyline (0.3 and 1 mg/kg, *n* = 16), moclobemide (3 mg/kg, *n* = 12) or sertraline (3 and 10 mg/kg, *n* = 12) 24 h before the choice test. Amitriptyline produced sustained attenuation of FG7142-induced negative bias 24 h after treatment (*F*_(2,30)_ = 7.081, *p* = 0.0030), with no bias observed at both 0.3 mg/kg (*t*_(15)_ = 0.5222, *p* = 0.6091) and 1 mg/kg (*t*_(11)_ = 1.820, *p* = 0.0960). A main effect of treatment was also observed for sertraline (*F*_(2,22)_ = 4.188, *p* = 0.0288) but only animals given 3 mg/kg differed from vehicle controls (*p* = 0.0215), while negative affective bias persisted at 1 mg/kg (*t*_(11)_ = 0.5180, *p* = 0.6147). Moclobemide failed to attenuate FG7142-induced negative bias when tested 24 h post-treatment (*t*_(11)_ = 0.5180, *p* = 0.6147), with 3 mg/kg treated animals exhibiting a negative bias similar to vehicle. Data are shown as mean % choice bias ± SEM and were analysed using a one-sample *t*-test (^***^p < 0.001, ^****^p < 0.0001) and Dunnett’s test following main effect with RM-ANOVA or two-tailed paired *t*-test (^#^*p* < 0.05, ^##^*p* < 0.01).

Amitriptyline and high dose sertraline showed sustained attenuation of negative biases 24 h post-treatment, while moclobemide had no sustained effects (Fig. 4). All vehicle-treated animals demonstrated a negative bias toward the FG7142-paired substrate 24 h after treatment and 48 h after the last pairing session. Animals treated with both 0.3 and 1 mg/kg amitriptyline showed sustained attenuation of the negative bias when tested 24 h post-treatment (*F*_(2,30)_ = 7.081, *p* = 0.0030, *η*^2^ = 0.321, *n* = 16), with no bias observed compared to a neutral 0% choice bias for both 0.3 mg/kg (one-sample *t*-test, *t*_(15)_ = 0.5222, *p* = 0.6091) and 1 mg/kg (one-sample *t*-test, *t*_(15)_ = 1.649, *p* = 0.1200). A main effect of treatment was observed with sertraline (*F*_(2,22)_ = 4.188, *p* = 0.0288, *η*^2^ = 0.276, *n* = 12) but only 3 mg/kg was significantly different from the vehicle group (Dunnett’s test, *p* = 0.0215), with the 1.0 mg group failing to exhibit a similar attenuating effect (Dunnett’s test, *p* = 0.7674, one-sample *t*-test, *t*_(11)_ = 1.820, *p* = 0.0960). Moclobemide treatment did not have an effect on the FG7142-induced negative bias when given 24 h prior to testing. There was no evidence of non-specific impairments during the choice test for any of the treatments or doses tested (*F* < 3.32, *p* > 0.05) (Supplementary Table S8a). Treatment with 3 mg/kg FG7142 also did not produce any such effects on performance during pairing sessions (Supplementary Table S8b).

## Discussion

This study provides new insights into the neuropsychological effects of CADs, suggesting that pharmacological differences within class may give rise to distinct effects in terms of affective bias modification. Amitriptyline (TCA) demonstrated the broadest effects, inducing a positive bias during new learning while also attenuating negatively biased memories both acutely and 24 h post-treatment. The effects on negative biases at retrieval were similar to those observed with the muscarinic antagonist and RAAD scopolamine, although likely involving lower levels of receptor occupancy given amitriptyline has weaker affinity for muscarinic receptors than scopolamine [29, 35]. At least behaviourally, these findings suggest a potential contribution of the muscarinic antagonism in the neuropsychological effects of amitriptyline. The MAOI moclobemide affected only new learning, whereas the SSRI sertraline showed highly dose-dependent effects, with only the 3 mg/kg dose modulating affective biases associated with both new learning and past experiences. Previous studies with the serotonin and noradrenaline reuptake inhibitor (SNRI) venlafaxine found effects limited to new learning, while the RAAD ketamine selectively modulated affective biases associated with past experiences [24, 29]. Taken together, these findings suggest that differences in antidepressant pharmacology shape their neuropsychological effects, which may in turn influence both efficacy and the time course of clinical outcomes.

Amitriptyline’s ability to modulate both encoding and retrieval processes is consistent with its pharmacological profile, namely inhibition of 5-HT/NA reuptake and antagonism of muscarinic acetylcholine (mACh) receptors [6, 36]. Scopolamine, a mACh antagonist, has been shown to improve symptoms in treatment-resistant depressed patients within a few days of daily administration [11, 37, 38]. In the ABT, scopolamine did not affect new learning but attenuated negatively biased memories both acutely and 24 h post-treatment [29, 35]. These findings suggest that anticholinergic mechanisms may contribute to amitriptyline’s antidepressant effects and may explain why early studies with TCAs reported clinical improvements after only a few days of treatment [8]. However, both amitriptyline and scopolamine differed from ketamine and psilocybin at 24 h as they did not facilitate re-learning of a negatively biased memory with a relatively more positive affective valence [4, 29, 35].

Sertraline and moclobemide both primarily modulate serotonin neurotransmission and, like other CADs tested in the ABT, acute treatment induced a positive affective bias in the new learning protocol. These effects were highly dose-dependent, with lower doses facilitating positive affective biases, whereas higher doses induced negative biases, a pattern previously observed with fluoxetine and citalopram [30]. Such dose-dependent effects may reflect the anxiogenic consequences of enhanced 5-HT signalling, which have also been reported in patients, particularly during early treatment [39, 40]. Although both sertraline and moclobemide promote serotonergic transmission, they act through distinct pharmacological mechanisms that may explain their differential effects on retrieval of negatively biased memories. SSRIs increase synaptic 5-HT by blocking its transporter [4, 41], whereas moclobemide inhibits enzymatic breakdown, producing a more gradual rise in 5-HT levels [42, 43]. The finding that sertraline, but not moclobemide, modulated negative bias retrieval suggests that direct transporter inhibition exerts more immediate effects on affective bias circuits. Like amitriptyline, sertraline’s modulation of affective biases linked to past experiences did not extend to re-learning effects at 24 h. This divergence indicates that not all drugs enhancing 5-HT availability exert equivalent effects on neuropsychological processes relevant to MDD. The dose-dependent effects we observed likely reflect the biphasic effects of enhanced serotonergic transmission in terms of affective state. At lower doses, moderate increases in synaptic 5-HT may facilitate positive emotional processing. However, excessive 5-HT signalling at higher doses may have an early anxiogenic effect contributing to the negative affective bias seen at higher doses. With chronic use, receptor adaptation may reduce the anxiogenic effects, potentially leading to improved efficacy. These findings also suggest that SSRIs may have a narrow dose range over which they are most effective at generating positive affective biases, and drugs acting at both serotonin and noradrenaline may be less likely to induce negative affective biases at higher doses.

Consistent with our findings with fluoxetine and citalopram, sertraline positively biased new experiences but with a relatively narrow dose-response relationship. We have not tested most of the CADs using the retrieval protocols but have previously shown that the mixed serotonin and noradrenaline re-uptake inhibitor, venlafaxine, does not attenuate biased memories [30, 33]. It will be interesting to see if the ability to modulate biased memories acutely is similarly observed with other SSRIs or if the effects vary within class and are related to other aspects of their pharmacology. Venlafaxine increases both 5-HT and NA levels [44], suggesting differing relative contributions of these neurotransmitters to affective bias modulation. Whereas NA is strongly implicated in attentional and arousal-related processes [45, 46], 5-HT appears to play a greater role in affective memory retrieval [47–49]. These differences highlight the potential importance of CAD interactions with clinically relevant neuropsychological processes and suggest that monoaminergic antidepressants may not act uniformly.

Single-dose administration was intentionally employed to isolate acute neuropsychological effects of CADs on affective bias. This approach was driven by an interest in comparing these results with previous ABT psychopharmacology and the acute studies performed in human subjects which led to the neuropsychological hypothesis of antidepressant efficacy. In this hypothesis, greater emphasis is placed on the acute neuropsychological effects and interaction with experience-dependent plasticity, learning, and memory [29]. In future, it would be interesting to assess how neuroadaptive changes under chronic administration and steady state levels of antidepressants influence these neuropsychological effects.

The results from the different ABT protocols show there is some variability in individual animal’s responses to acute antidepressant treatments. This has not been explored further, but future studies could investigate if different individuals show consistent heterogeneity in affective bias modification and whether this is stable within antidepressant class or suggests individual subjects respond in different ways, potentially enabling more personalised treatment strategies.

The RLA is used to assess whether the antidepressant effects on affective biases observed in the ABT reflect modifications in emotional processing rather than non-specific effects on memory retrieval [31]. At doses that attenuated negative affective biases, neither amitriptyline nor sertraline influenced the retrieval of a reward-induced bias, confirming their specificity in targeting emotional circuits (Supplementary Tables S5–S8). This specificity underscores the potential of these drugs to effectively target the cognitive–affective disruptions characteristic of MDD and to provide therapeutic benefits that directly address core features of the disorder.

Taken together, these findings further support the neuropsychological hypothesis of antidepressant action, which posits that antidepressants exert therapeutic effects by shifting affective biases prior to overt mood improvement [5, 50, 51]. We have previously suggested that the capacity to modulate affective biases associated with retrieval of past experiences is related to the rate of onset of clinical benefits and, where a treatment affects more than one aspect of affective bias modulation, this may lead to both rapid and enduring antidepressant effects as is seen with the psychedelic psilocybin.

Traditionally, CADs were assumed to act through delayed neuroadaptive mechanisms [52, 53]; however, the present results support patient and experimental medicine studies that show CADs produce measurable neuropsychological effects within hours of administration. Within this framework, the delayed onset of action has been attributed to the time required for new, positively biased experiences to accumulate and influence subjective mood [5, 50, 51]. The capacity of RAADs to modulate negative biases associated with past experiences may enable a more rapid shift in mood [24, 29], although these data indicate that distinctions between classes may be less clear-cut than previously assumed. Integrating these findings with prior studies using the ABT, we propose the existence of at least three core forms of affective bias modification relevant to antidepressant efficacy. Drugs that bias only new experiences are likely to demonstrate delayed onset of action and require chronic treatment to enhance positive affective processing and improve mood [5, 24, 29, 30]. Drugs that attenuate negative biases without facilitating re-learning with a more positive affective valence may act more rapidly but, in the short term, may primarily induce neutral emotional processing or potentially emotional blunting [24, 29]. These findings suggest different underlying neuropsychological mechanism where emotional processing only shifts to neutral rather than positive. It remains to be further investigated but our prediction is that emotional blunting may induce this type of outcome i.e. the memory retrieval is neither positive nor negative.

In contrast, drugs that facilitate a change in affective bias e.g. post-treatment retrieval with a relatively more positive affective valence, such as ketamine and psilocybin, can produce rapid and sustained effects after a single dose [24, 29, 54]. The findings with the TCA amitriptyline could explain why early clinical studies observe beneficial effects after a few days of treatment and suggest the onset of action of drugs such as venlafaxine may be accelerated if initially co-administered with a muscarinic antagonist [55, 56].

From a clinical perspective, these findings challenge the assumption that all CADs exert similar efficacy through a shared underlying biochemical mechanism [57, 58]. Differences among SSRIs, TCAs, and MAOIs in their effects on neuropsychological processes relevant to MDD suggest that treatment efficacy and duration may be mediated by distinct neuropsychological effects and underlying neural mechanisms. Amitriptyline’s broad modulation of affective biases, particularly its sustained attenuation of negative biases, is consistent with meta-analytic evidence indicating that it is among the most effective antidepressants [59]. Its combined 5-HT and NA reuptake inhibition, together with mACh receptor antagonism, may underlie this broader modulation of affective bias circuits compared with SSRIs, which primarily act by enhancing 5-HT availability.

In conclusion, this study demonstrates that CADs do not exert uniform effects on affective biases but instead show important variations in their influence on new experiences versus biased memories. The acute effects observed in the ABT indicate that all CADs act on cognitive-affective mechanisms prior to the emergence of overt clinical improvements. As cognitive-affective shifts are increasingly recognised as early biomarkers of antidepressant efficacy [15], understanding how CADs modulate affective processing may help refine treatment strategies. By identifying drugs that influence affective biases at different stages, future research may clarify which antidepressants are most effective for specific cognitive-affective profiles. Furthermore, investigating whether combining CADs with RAADs that facilitate re-learning and cognitive flexibility enhances treatment efficacy could further optimise therapeutic outcomes for patients with MDD.

## Supplementary information

Supplementary materials and methods

Data All

## Data Availability

All data supporting the findings of this study are included in the published article and its supplementary information files.
