# Supplementary material for: Comparing affective bias modification by first- and second-generation antidepressants in male rats using a translational behavioural task
Source: Neuropsychopharmacology. 2026 Feb 26;51(6):1056–64. doi: 10.1038/s41386-026-02376-4 (PMC13125249; doi:10.1038/s41386-026-02376-4)
Supplement: Supplementary file 1 — Supplementary materials and methods [file 41386_2026_2376_MOESM1_ESM.docx]

**Comparing affective bias modification by first- and second-generation antidepressants in male rats using a translational behavioural task**

Katie A Kamenish^1^, Emma N Cahill^1^, Emma S J Robinson^1*^

**Supplementary materials**

**Supplementary Figures**

*Figure S1*

**
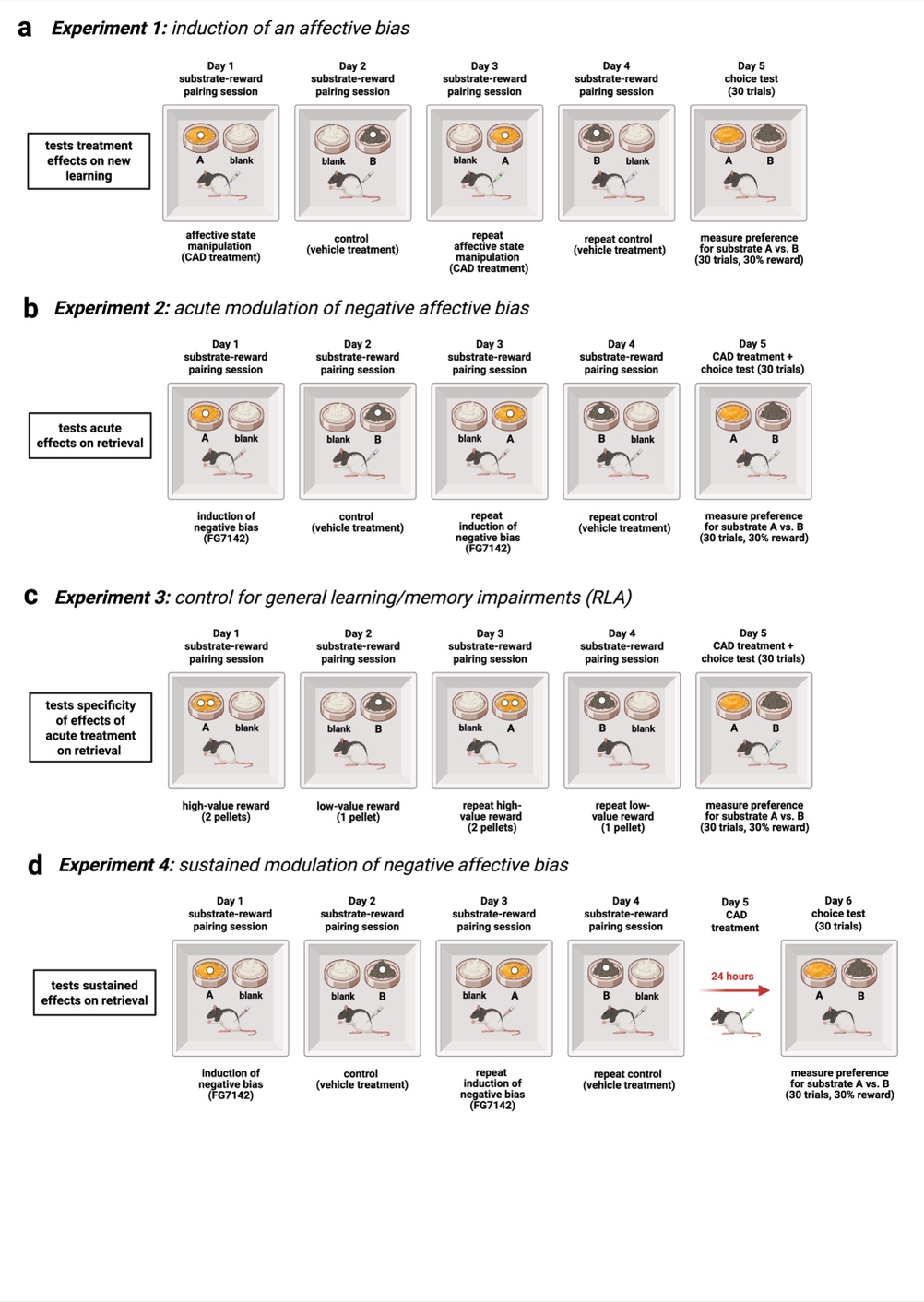
**

**Figure S1:** Overview of ABT and RLA protocols comparing CAD classes on affective bias modulation. The ABT measures affective biases in rodents by creating two reward memories under an affective state manipulation or neutral/control state. This assay posits that affective bias changes perceived reward value, gauged by preferences for substrate-reward associations learned during affective versus neutral states. In the task, animals associate a digging substrate with a food reward. The reward value of each experience is kept equal as animals undergo four pairing sessions across four days followed by a choice test presenting both substrates, and choice bias is assessed over 30 randomly reinforced trials. Panel **a** outlines the protocol for assessing new learning effects, with treatments administered prior to pairing sessions and affective bias quantified at retrieval during choice testing. Panel **b** shows the protocol for assessing acute attenuation of established negative bias by CADs, where a negative bias is first generated during pairing sessions by administration of FG7142, then the CAD is administered before the choice test. Treatments showing acute negative bias attenuation are further tested with the RLA protocol in panel **c** to confirm if ABT effects are specific to affective state modulation and not due to non-specific reward learning impairments. A positive bias is generated by varying reward value (one versus two pellets) pairings. CAD treatments are then administered before the choice test to measure impact on retrieval of reward-induced bias. In absence of any non-specific effects, animals proceed with the ABT protocol in panel **d**. Following negative bias induction by FG7142, treatments are administered 24 hours before the choice test to evaluate sustained negative bias modulation. Created in BioRender. Kamenish, K. (2025) https://BioRender.com/r770b6n

*Figure S2*

**
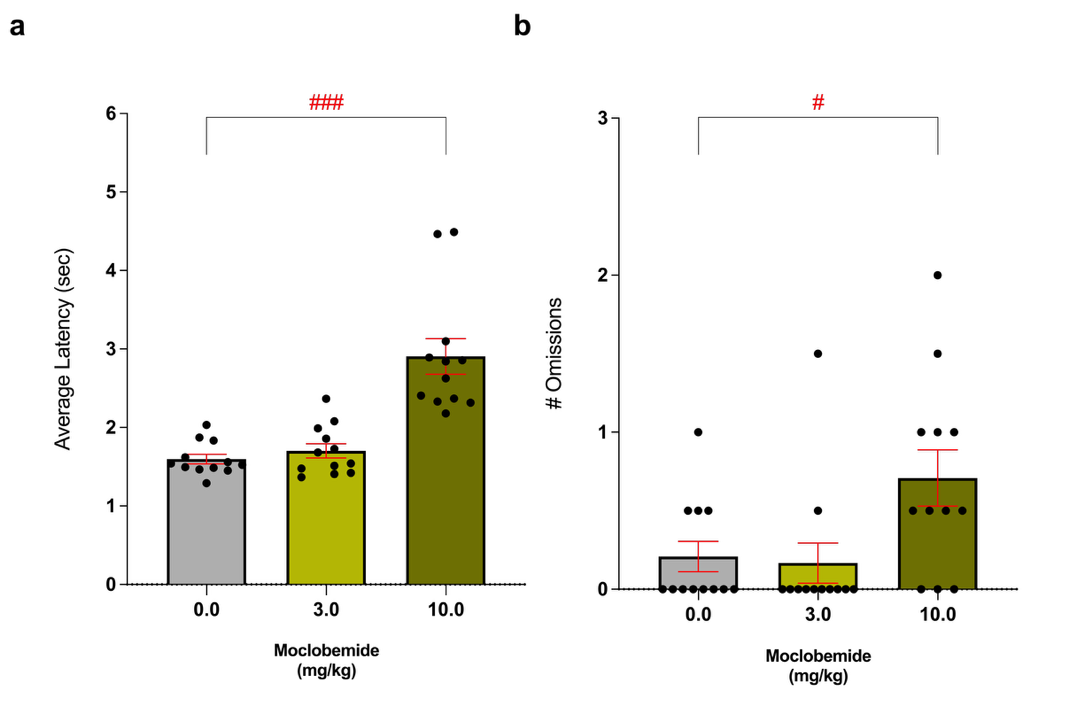
**

**Figure S2:** Moclobemide produced non-specific effects on task performance during substrate-reward pairing sessions. When administered prior to pairing sessions, moclobemide (3 and 10 mg/kg, n=12) significantly increased latency to respond during pairing trials (F_(2,22)_=23.29, p=0.0003) but only at the highest dose of 10 mg/kg (p=0.0005) and not at 3 mg/kg (p=0.3370). There was an additional main effect of moclobemide treatment on number of omitted trials (F_(2,22)_=6.280, p=0.0070), but this was again only observed at 10 mg/kg (p=0.0142) and not at the lower dose (p=0.9571). These findings indicate that the negative bias observed following treatment with 10 mg/kg moclobemide may have been influenced by these impairments of task performance and therefore cannot be interpreted as the result of a specific affective state modulation. Data are shown as mean % choice bias ± SEM and were analyzed using Dunnett’s test following main effect with RM-ANOVA (^*^p < 0.05,^**^p < 0.01).

**Supplementary Tables**

*Table S1*

| **Cohort** | **Test** | **Substrate A** | **Substrate B** | **Substrate C (blank)** |
| --- | --- | --- | --- | --- |
| **Cohort 1** | **Test 1** | cotton wool balls | hairbands | stringy cloth |
|  | **Test 2** | yellow bath sponge | duster | absorbent fibre |
|  | **Test 3** | shredded paper | silk | organza |
|  | **Test 4** | tights | umbrella | Christmas ribbon |
|  | **Test 5** | balloons | leather | cotton mix |
|  | **Test 6** | newspaper | paper pet bedding | paper chad |
|  | **Test 7** | string | cardboard | black satin |
|  | **Test 8** | felt | exfoliating gloves | dishcloth |
|  | **Test 9** | purple gift ribbon | raffia ribbon | pompoms |
|  | **Test 10** | suede | fleece | chenille |
|  | **Test 11** | crepe paper | scarf yarn | sparkly fibre |
| **Cohort 2** | **Test 1** | tissue paper | absorbent fibre | foam shapes |
|  | **Test 2** | hessian sack | brown pet bedding | cork |
|  | **Test 3** | corrugated paper | cellulose sponge | perlite |
|  | **Test 4** | pompoms | fur | polyester |
|  | **Test 5** | straws | inner tube | plastic scourer |
|  | **Test 6** | cotton mix | silk | towel |
|  | **Test 7** | foam padding | denim | backpack strap |
|  | **Test 8** | partition paper | tablecloth | cardigan |
|  | **Test 9** | aspen | matchsticks | hemp |
|  | **Test 10** | shoelaces | wool | sisal twine |
| **Cohort 3** | **Test 1** | straw hat | bath mat | pink cowboy hat |
|  | **Test 2** | yellow cord | towel | pipe cleaners |
|  | **Test 3** | poster | sequins | buttons |
|  | **Test 4** | tissue paper | hessian sack | brown pet bedding |
|  | **Test 5** | fur | felt | tights |
|  | **Test 6** | hairbands | suede | organza |
|  | **Test 7** | fleece | raffia ribbon | sparkly fibre |

**Table S1:** List of substrates used in experiments for all cohorts.

*Table S2*

| **Day** | **1** | **2** | **3** | **4** | **5/6** |
| --- | --- | --- | --- | --- | --- |
| **Subject** | **Pairing 1** | **Pairing 2** | **Pairing 3** | **Pairing 4** | **Choice Test** |
| **Rat 1** | A vs. blank  **DRUG** | B vs. blank  **VEHICLE** | A vs. blank  **DRUG** | B vs. blank  **VEHICLE** | A vs. blank  **30 TRIALS** |
| **Rat 2** | B vs. blank **DRUG** | A vs. blank  **VEHICLE** | B vs. blank  **DRUG** | A vs. blank  **VEHICLE** | A vs. blank  **30 TRIALS** |
| **Rat 3** | A vs. blank  **VEHICLE** | B vs. blank  **DRUG** | A vs. blank  **VEHICLE** | B vs. blank  **DRUG** | A vs. blank  **30 TRIALS** |
| **Rat 4** | B vs. blank  **VEHICLE** | A vs. blank  **DRUG** | B vs. blank  **VEHICLE** | A vs. blank  **DRUG** | A vs. blank  **30 TRIALS** |

**Table S2:** Standard protocol for experiments testing drug-induced affective bias against vehicle. Animals receive drug treatment or vehicle counterbalanced over four substrate-reward pairing sessions. Substrate (reward-paired substrates - ‘A’ or ‘B’ versus unrewarded substrate - ‘blank’) and pairing day are also counter-balanced to produce four groups.

*Table S3*

| **Day** | **1** | **2** | **3** | **4** | **5/6** |
| --- | --- | --- | --- | --- | --- |
| **Subject** | **Pairing 1** | **Pairing 2** | **Pairing 3** | **Pairing 4** | **Choice Test** |
| **Rat 1** | A vs. blank  **1 PELLET** | B vs. blank  **2 PELLETS** | A vs. blank  **1 PELLET** | B vs. blank  **2 PELLETS** | A vs. B  **30 TRIALS** |
| **Rat 2** | B vs. blank  **1 PELLET** | A vs. blank  **2 PELLETS** | B vs. blank  **1 PELLET** | A vs. blank  **2 PELLETS** | A vs. B  **30 TRIALS** |
| **Rat 3** | A vs. blank  **2 PELLETS** | B vs. blank  **1 PELLET** | A vs. blank  **2 PELLETS** | B vs. blank  **1 PELLET** | A vs. B  **30 TRIALS** |
| **Rat 4** | B vs. blank  **2 PELLETS** | A vs. blank  **1 PELLET** | B vs. blank  **2 PELLETS** | A vs. blank  **1 PELLET** | A vs. B  **30 TRIALS** |

**Table S3:** Standard protocol for experiments testing reward-induced bias in the RLA. Animals receive a high-value (two pellets) or low-value (one pellet) reward counterbalanced over four substrate-reward pairing sessions. Substrate (reward-paired substrates - ‘A’ or ‘B’ versus unrewarded substrate - ‘blank’) and pairing day are also counter-balanced to produce four groups.

*Table S4*

| **Cohort** | **Treatment** | **Dose (mg/kg)** | **Route of Admin.** | **Pretreatment Times** |
| --- | --- | --- | --- | --- |
| 1 | **Amitriptyline** | 0.0, 0.3, 1.0, 3.0 | oral admin. in palatable solution | 2 hrs. / 24 hrs. |
| 1 | **Venlafaxine** | 0.0, 3.0 | oral admin. in palatable solution | 2 hrs. |
| 2,3 | **Sertraline** | 0.0, 1.0, 3.0, 10.0 | oral admin. in palatable solution | 2 hrs. / 24 hrs. |
| 3 | **Moclobemide** | 0.0, 3.0, 10.0 | subcutaneous injection | 30 mins. / 24 hrs. |
| 1,2,3 | **FG7142** | 0.0, 3.0 | subcutaneous injection | 30 mins. |

**Table S4:** Summary of drug treatments for all cohorts.

*Table S5*

| **Treatment (mg/kg)** | | **Trials to Criterion** | | **Response Latency (sec.)** | | **Omissions** | |
| --- | --- | --- | --- | --- | --- | --- | --- |
|  |  | **Vehicle** | **Drug** | **Vehicle** | **Drug** | **Vehicle** | **Drug** |
| **Amitriptyline** | 0.0 | 7.2 ± 0.1 | 6.8 ± 0.2 | 1.8 ± 0.0 | 2.0 ± 0.1 | 0.1 ± 0.1 | 0.3 ± 0.1 |
|  | 0.3 | 7.6 ± 0.3 | 7.0 ± 0.2 | 1.4 ± 0.1 | 1.9 ± 0.1 | 0.5 ± 0.1 | 0.3 ± 0.1 |
|  | 1.0 | 7.1 ± 0.1 | 7.1 ± 0.3 | 2.0 ± 0.0 | 1.9 ± 0.1 | 0.3 ± 0.1 | 0.2 ± 0.1 |
|  | 3.0 | 7.0 ± 0.2 | 7.1 ± 0.3 | 1.4 ± 0.1 | 1.7 ± 0.1 | 0.4 ± 0.2 | 0.2 ± 0.1 |
| **Venlafaxine** | 3.0 | 7.6 ± 0.3 | 7.6 ± 0.2 | 2.0 ± 0.1 | 1.9 ± 0.1 | 0.3 ± 0.1 | 0.2 ± 0.1 |
| **Moclobemide** | 0.0 | 6.9 ± 0.2 | 7.1 ± 0.2 | 1.5 ± 0.0 | 1.6 ± 0.1 | 0.2 ± 0.1 | 0.2 ± 0.1 |
|  | 3.0 | 7.4 ± 0.2 | 7.5 ± 0.2 | 1.9 ± 0.0 | 1.7 ± 0.1 | 0.2 ± 0.1 | 0.2 ± 0.1 |
|  | 10.0 | 7.1 ± 0.2 | 7.5± 0.3 | 1.4 ± 0.1 | 2.9 ± 0.2^**^ | 0.2 ± 0.1 | 0.7 ± 0.2^*^ |
| **Sertraline** | 0.0 | 6.9 ± 0.2 | 7.1 ± 0.3 | 1.9 ± 0.1 | 1.4 ± 0.1 | 0.1 ± 0.2 | 0.2 ± 0.1 |
|  | 1.0 | 6.8 ± 0.3 | 7.5 ± 0.2 | 1.6 ± 0.1 | 1.4 ± 0.1 | 0.3 ± 0.2 | 0.1 ± 0.1 |
|  | 3.0 | 6.8 ± 0.2 | 6.9 ± 0.3 | 1.8 ± 0.1 | 1.4 ± 0.1 | 0.2 ± 0.1 | 0.3 ± 0.1 |
|  | 10.0 | 6.9 ± 0.3 | 6.9 ± 0.2 | 1.5 ± 0.1 | 1.5 ± 0.1 | 0.2 ± 0.1 | 0.4± 0.2 |

**Table S5:** Pairing session data for ABT experiments assessing new learning effects. Number of trials to criterion, latency to respond, and number of omitted trials were recorded for each pairing session. Data shown as mean (n=12-16 animals/group) ± SEM averaged from pairing sessions for each group. There were no significant effects of amitriptyline or sertraline during pairing sessions, but 10 mg/kg moclobemide significantly increased latency to respond and number of omitted trials (^*^p < 0.05,^**^p < 0.01; for details see *Fig. S2*).

*Table S6a*

| **Treatment** | **Dose (mg/kg)** | **Response Latency (sec.)** | **Omissions** |
| --- | --- | --- | --- |
| **Amitriptyline** | 0.0 | 1.5 ± 0.1 | 0.4 ± 0.2 |
|  | 0.3 | 1.4 ± 0.1 | 0.3 ± 0.1 |
|  | 1.0 | 1.4 ± 0.1 | 0.6 ± 0.3 |
| **Moclobemide** | 0.0 | 1.5 ± 0.0 | 0.6 ± 0.2 |
|  | 3.0 | 1.5 ± 0.0 | 0.3 ± 0.1 |
| **Sertraline** | 0.0 | 1.5 ± 0.1 | 0.2 ± 0.1 |
|  | 3.0 | 1.6 ± 0.1 | 0.2 ± 0.1 |
|  | 10.0 | 1.5 ± 0.1 | 0.4 ± 0.2 |

**Table S6a:** Choice test data for ABT experiments assessing acute negative bias modulation. Latency to respond and number of omitted trials were recorded for each animal during 30 choice test trials. Data shown as mean (n=12-16 animals/group) ± SEM averaged from individuals’ data for each group. None of the treatments produced significant effects during choice testing.

*Table S6b*

| **Treatment (mg/kg)** | | **Trials to Criterion** | | **Response Latency (sec.)** | | **Omissions** | |
| --- | --- | --- | --- | --- | --- | --- | --- |
|  |  | **Vehicle** | **FG7142** | **Vehicle** | **FG7142** | **Vehicle** | **FG7142** |
| **Amitriptyline** | 0.0 | 6.7 ± 0.2 | 7.3 ± 0.3 | 1.7 ± 0.1 | 1.7 ± 0.1 | 0.3 ± 0.1 | 0.9 ± 0.2 |
|  | 0.3 | 6.4 ± 0.1 | 6.8 ± 0.3 | 1.6 ± 0.1 | 1.6 ± 0.1 | 0.8 ± 0.2 | 0.3 ± 0.1 |
|  | 1.0 | 7.3 ± 0.2 | 6.7 ± 0.2 | 1.7 ± 0.1 | 1.6 ± 0.1 | 0.7 ± 0.3 | 0.5 ± 0.1 |
| **Moclobemide** | 0.0 | 7.8 ± 0.3 | 7.1 ± 0.3 | 1.6 ± 0.04 | 1.5 ± 0.04 | 0.5 ± 0.1 | 0.3 ± 0.1 |
|  | 3.0 | 7.0 ± 0.2 | 7.3 ± 0.2 | 1.6 ± 0.05 | 1.5 ± 0.03 | 0.2 ± 0.1 | 0.4 ± 0.1 |
| **Sertraline** | 0.0 | 7.6 ± 0.3 | 7.7 ± 0.2 | 1.4 ± 0.1 | 1.4 ± 0.07 | 0.3 ± 0.1 | 0.2 ± 0.1 |
|  | 3.0 | 7.0 ± 0.2 | 8.0 ± 0.4 | 1.5 ± 0.07 | 1.6 ± 0.1 | 0.2 ± 0.1 | 0.2 ± 0.1 |
|  | 10.0 | 7.8 ± 0.3 | 7.3 ± 0.3 | 1.6 ± 0.1 | 1.5 ± 0.1 | 0.2 ± 0.1 | 0.1 ± 0.06 |

**Table S6b:** Pairing session data for ABT experiments assessing acute negative bias modulation. Number of trials to criterion, latency to respond, and number of omitted trials were recorded for each pairing session. Data shown as mean (n=12-16 animals/group) ± SEM averaged from pairing sessions for each group (vehicle versus FG7142). Administration of FG7142 before pairing sessions did not produce any significant effects on task performance in all groups.

*Table S7*

| **Treatment** | **Dose (mg/kg)** | **Response Latency (sec.)** | **Omissions** |
| --- | --- | --- | --- |
| **Amitriptyline** | 0.0 | 1.4 ± 0.1 | 0.6 ± 0.3 |
|  | 0.3 | 1.4 ± 0.0 | 0.9 ± 0.3 |
|  | 1.0 | 1.4 ± 0.0 | 0.5 ± 0.2 |
| **Sertraline** | 0.0 | 1.2 ± 0.0 | 0.2 ± 0.1 |
|  | 3.0 | 1.3 ± 0.0 | 0.3 ± 0.1 |

**Table S7:** Choice test data for RLA experiments assessing specificity of effects on affective bias. Latency to respond and number of omitted trials were recorded for each animal during 30 choice test trials. Data shown as mean (n=12-16 animals/group) ± SEM averaged from individuals’ data for each group. None of the treatments produced significant effects during choice testing.

*Table S8a*

| **Treatment** | **Dose (mg/kg)** | **Response Latency (sec.)** | **Omissions** |
| --- | --- | --- | --- |
| **Amitriptyline** | 0.0 | 1.6 ± 0.1 | 0.8 ± 0.4 |
|  | 0.3 | 1.4 ± 0.0 | 0.6 ± 0.2 |
|  | 1.0 | 1.5 ± 0.1 | 0.6 ± 0.2 |
| **Moclobemide** | 0.0 | 1.6 ± 0.1 | 0.5 ± 0.2 |
|  | 3.0 | 1.5 ± 0.0 | 0.3 ± 0.2 |
| **Sertraline** | 0.0 | 1.2 ± 0.0 | 0.2 ± 0.1 |
|  | 1.0 | 1.2 ± 0.0 | 0.3 ± 0.2 |
|  | 3.0 | 1.2 ± 0.0 | 0.3 ± 0.2 |

**Table S8a:** Choice test data for ABT experiments assessing sustained negative bias modulation. Latency to respond and number of omitted trials were recorded for each animal during 30 choice test trials. Data shown as mean (n=12-16 animals/group) ± SEM averaged from individuals’ data for each group. None of the treatments produced significant effects during choice testing.

*Table S8b*

| **Treatment (mg/kg)** | | **Trials to Criterion** | | **Response Latency (sec.)** | | **Omissions** | |
| --- | --- | --- | --- | --- | --- | --- | --- |
|  |  | **Vehicle** | **FG7142** | **Vehicle** | **FG7142** | **Vehicle** | **FG7142** |
| **Amitriptyline** | 0.0 | 6.9 ± 0.2 | 7.2 ± 0.2 | 1.6 ± 0.1 | 1.8 ± 0.1 | 0.3 ± 0.2 | 0.6 ± 0.2 |
|  | 0.3 | 7.2 ± 0.2 | 6.9 ± 0.2 | 1.6 ± 0.05 | 1.5 ± 0.06 | 0.4 ± 0.1 | 0.5 ± 0.1 |
|  | 1.0 | 6.8 ± 0.2 | 7.2 ± 0.3 | 1.7 ± 0.07 | 1.6 ± 0.04 | 0.7 ± 0.2 | 0.8 ± 0.1 |
| **Moclobemide** | 0.0 | 7.3 ± 0.3 | 7.6 ± 0.2 | 1.6 ± 0.08 | 1.6 ± 0.1 | 0.5 ± 0.1 | 0.5 ± 0.1 |
|  | 3.0 | 7.1 ± 0.3 | 7.6 ± 0.3 | 1.7 ± 0.05 | 1.7 ± 0.06 | 0.5 ± 0.2 | 0.4 ± 0.2 |
| **Sertraline** | 0.0 | 6.8 ± 0.2 | 6.9 ± 0.2 | 1.2 ± 0.04 | 1.2 ± 0.03 | 0.1 ± 0.05 | 0.1 ± 0.07 |
|  | 1.0 | 7.3 ± 0.2 | 6.6 ± 0.2 | 1.2 ± 0.03 | 1.2 ± 0.03 | 0.2 ± 0.07 | 0.2 ± 0.1 |
|  | 3.0 | 6.6 ± 0.2 | 6.8 ± 0.1 | 1.2 ± 0.03 | 1.2 ± 0.05 | 0.0± 0.0 | 0.2 ± 0.07 |

**Table S8b:** Pairing session data for ABT experiments assessing sustained negative bias modulation. Number of trials to criterion, latency to respond, and number of omitted trials were recorded for each pairing session. Data shown as mean (n=12-16 animals/group) ± SEM averaged from pairing sessions for each group (vehicle versus FG7142). Administration of FG7142 before pairing sessions did not produce any significant effects on task performance in all groups.
